# Genomic signatures of adaptive introgression and environmental adaptation in the Sheko cattle of southwest Ethiopia

**DOI:** 10.1371/journal.pone.0202479

**Published:** 2018-08-16

**Authors:** Hussain Bahbahani, Arwa Afana, David Wragg

**Affiliations:** 1 Department of Biological Sciences, Faculty of Science, Kuwait University, Kuwait City, Kuwait; 2 Centre for Tropical Livestock Genetics and Health, The Roslin Institute, Edinburgh, United Kingdom; Institute of Animal Sciences, GERMANY

## Abstract

Although classified as an African taurine breed, the genomes of Sheko cattle are an admixture of Asian zebu and African taurine ancestries. They populate the humid Bench Maji zone in Sheko and Bench districts in the south-western part of Ethiopia and are considered as a trypanotolerant breed with high potential for dairy production. Here, we investigate the genome of Sheko cattle for candidate signatures of adaptive introgression and positive selection using medium density genome-wide SNP data. Following locus-ancestry deviation analysis, 15 and 72 genome regions show substantial excess and deficiency in Asian zebu ancestry, respectively. Nine and 23 regions show candidate signatures of positive selection following extended haplotype homozygosity (EHH)-based analyses (*iHS* and *Rsb)*, respectively. The results support natural selection before admixture for one *iHS*, one *Rsb* and three zebu ancestry-deficient regions. Genes and/or QTL associated with bovine immunity, fertility, heat tolerance, trypanotolerance and lactation are present within candidate selected regions. The identification of candidate regions under selection in Sheko cattle warrants further investigation of a larger sample size using full genome sequence data to better characterise the underlying haplotypes. The results can then support informative genomic breeding programmes to sustainably enhance livestock productivity in East African trypanosomosis infested areas.

## Introduction

The history of cattle in Africa began with the migration of humpless *Bos taurus taurus* (taurine) from their center of domestication in the Near East to the African continent through Egypt about 5000 years BC [1]. It was followed by the introduction of *Bos taurus indicus* (indicine or zebu) from their center(s) of domestication on the Indian subcontinent [2] around 2000 years BC, with further zebu arriving around 700 years AD following Arabs trading along the East coast of Africa, and the onset of the Swahili civilization [3].

Given the sole presence of taurine mitochondrial DNA haplogroups in African cattle [4, 5], together with zebu-specific *Y* chromosome alleles [6], a male-mediated pattern of zebu introgression to the continent is the favored hypothesis [6]. Following the African rinderpest epidemic at the end of the 19^th^ century, which led to a massive eradication of susceptible African taurine cattle, dispersal of the more resistant zebu ancestry was accelerated in the western and southern parts of the continent [3, 7].

Presently there are more than 150 recognized African cattle breeds or populations, classified as either taurine, zebu, sanga (an ancient stabilized taurine x zebu crossbreed), or a sanga x zebu crossbreed called zenga [8]. Genetically, most of the African cattle are admixed populations of zebu x taurine ancestries with a gradient of indicine ancestry peaking amongst the East African breeds and declining westward and southward to reach its minimum level in West African cattle [3, 7]. Several African trypanolerant taurine cattle with little or no zebu ancestry still populate the highly tsetse fly (the vector of trypanosomosis) infested zones of West Africa (e.g. N’Dama in Guinea and Muturu in Nigeria) [9]. A possible ancient introgression of the extinct African auroch *Bos primigenius opisthonomus* within African cattle has been suggested [7] however this requires further investigation.

Sheko cattle are indigenous to East Africa inhabiting the humid Bench Maji zone mainly in Sheko and Bench districts at the south-western part of Ethiopia [10, 11]. They were originally classified as taurine, while recent genetic analyses indicates they are more of a sanga type with African taurine and Asian zebu genetic ancestry proportions of 0.3 ± 0.014 and 0.7 ± 0.014, respectively [12]. The presence of a small cervicothoracic hump in them alludes to their zebu ancestry [11]. Sheko cattle are adapted to these highly tsetse-infested areas and are considered trypanotolerant [11, 13, 14]. They also have good potential as dairy cattle for Africa, having large teats and the ability to yield on average 2.79 ± 0.06 liters of milk daily and 850.6 ± 24.16 liters per lactation period, which is 307.69 ± 6.13 days, depending on the on-farm management practice [11, 15].

Previous studies of East African shorthorn zebu [16, 17], Butana and Kenana zebu from Sudan [18], and taurine and zebu cattle breeds from the western and eastern parts of the African continent [19] have identified signatures of positive selection in genes and quantitative trait loci (QTL) associated with adaptive traits. Many of the genes and QTL identified were found to be involved in biological pathways, such as bovine immunity, reproduction, heat tolerance and coat color. These studies applied genome-wide analyses of genotype data generated using the Illumina BovineSNP50 Beadchip [16], the Illumina BovineHD BeadChip [17, 18], as well as full genome sequence data [17, 19]. In commercial cattle breeds genes associated with milk yield and composition, muscle development and coat color have also been identified to be under positive selection [20–22].

In admixed populations, large deviations in genomic local ancestry relative to the average genome-wide admixture level may represent possible adaptive introgression [22–24], particularly if these regions are of large size and/or overlap with candidate footprints of positive selection. In humans this approach has been previously used to define positively selected genomic regions in an admixed Puerto Rican population with local ancestry deviation in the human leukocyte antigen regions on chromosomes 6, 8 and 11 [23]. A later study on a population of African–American descent identified genomic regions with significant excess of African ancestry in genes linked to the onset of diabetes, pancreatic and lung cancer [24].

Analysis of the dairy/beef dual purpose Simmental x Red Holstein-Frisian admixed Swiss Fleckvieh cattle has revealed recent responses to selection using medium density genome-wide SNP data [22]. Two regions on BTA 13 and 18 showed significant local ancestry deviation towards Simmental ancestry. These regions carry genes associated with bovine fertility (*NKD1* and *NOD2*) and the *FTO* gene, which has a pleiotropic effect being involved in milk composition and fertility.

In this study, we employ genotype data generated using Illumina’s BovineSNP50 BeadChip to assess whether or not the genomic landscape of Sheko cattle has been under selection following introgression with zebu. We identify genomic regions in Sheko cattle with substantial locus-ancestry deviation and unusual extended haplotype homozygosity (EHH) and discriminate between the pre- and post-admixture selection pressures on the genome.

## Materials and methods

### SNP genotyping and quality control

Genome-wide SNP genotype data from the Illumina BovineSNP50 BeadChip version 1 [25] for 20 East African taurine Sheko, 25 West African taurine N’Dama and 21 Asian zebu Nelore cattle were obtained from the Bovine HapMap consortium [26]. Quality control analyses were carried out on 54,334 autosomal SNPs mapped to the UMD3.1 bovine reference genome using the *check*.*marker* function of the GenABEL package [27] for R software version 3.2.2 [28]. In total, 19,417 SNPs with minor allele frequency less than 0.05 and 6,886 SNPs with call rate less than 0.95 were removed. Among these, 5,766 SNPs failed both criteria, leaving 33,797 SNPs for downstream analyses. None of the samples had a SNP call rate < 0.95 or identity-by-state (IBS) > 0.95.

### Locus-ancestry deviation analysis

The Asian zebu and African taurine ancestry proportions were estimated in 1 Mb sliding genomic windows using the PCAdmix software version 1.0 [29]. fastPHASE software version 1.4 [30] was used to phase the genotyped SNPs into the corresponding haplotypes using K10 and T10 criteria. Population label information was provided to estimate the phased haplotype background. The Asian zebu ancestry proportion of each genomic window was estimated as the proportion of zebu haplotypes carried by the Sheko samples in that window. Windows deviating by two standard deviations (SD) from the mean zebu ancestry of all the genomic windows were considered as candidate regions with substantial excess/deficiency in Asian zebu ancestry.

### Extended haplotypes homozygosity (EHH)-derived statistics (Rsb and iHS)

Intra-population *iHS* [31] and inter-population *Rsb* [32] analyses were conducted using the rehh package [33] for R software to define candidate genomic regions with signatures of positive selection. The *iHS* analysis was carried out using genotyped SNPs with intra-population minor allele frequency ≥ 0.05. The *iHS* statistic is calculated by first defining the integral of the observed decay of EHH against the physical genomic position of the SNP, as one moves away from a core SNP for both the reference and alternative alleles until it reaches an arbitrary value of 0.05. These integrals, which are summed over both directions from the core SNP, are called *iHH*_*Ref*_ and *iHH*_*Alt*_ for the reference and alternative alleles, respectively. The natural log of the ratio (*iHH*_*Ref*_*/iHH*_*Alt*_) is standardized to generate an *iHS* value for each SNP with a mean 0 and variance 1, as described in Voight, Kudaravalli (31). As the standardized *iHS* values are normally distributed "Panel A in S1 Fig" and signatures of selection on both the reference and alternative alleles are equally important, a two-tailed Z test was applied to identify statistically significant SNPs. Two-sided *P*-values were derived as–log_10_(1–2|Φ(*iHS*)-0.5*|)*, where Φ*(iHS)* represents the Gaussian cumulative distribution function. Inter-population *Rsb* analyses [32] were conducted separately between Sheko cattle and African taurine (N’Dama) and Asian zebu (Nelore) cattle. In the *Rsb* analysis, the EHH for the two alleles of a SNP was averaged and weighted by their squared allele frequencies, which provided the site-specific EHH (EHHS). As with EHH, the observed decay of EHHS for each core SNP was integrated and summed over both directions in both populations (iES). An *Rsb* value for each SNP was obtained by standardizing the natural log ratio between the *iES* of Sheko population (*iES*_*Sheko*_) with iES of the second reference population (*iES*_*Reference*_), as described in Tang, Thornton (32). As the standardized *Rsb* values are normally distributed " Panels B and C in S1 Fig" a one-tailed Z-test was applied to identify statistically significant SNPs under selection in Sheko cattle (positive *Rsb* value). One-sided *P*-values were derived as–log_10_(1- Φ(*Rsb*)), where Φ*(Rsb)* represents the Gaussian cumulative distribution function. In both *iHS* and *Rsb*, -log_10_ (*P*-value) = 3, equivalent to a *P*-value of 0.001, was used as a threshold to define significant *iHS* and *Rsb* values. A candidate region was defined if at least two SNPs not separated by more than 500 Kb passed the significant threshold as followed by [16], which is the extent of linkage disequilibrium determined in the genomes of different taurine and indicine breeds [34].

### Functional characterization of the candidate regions

Genes mapped on the UMD3.1 reference bovine genome within substantial excess/deficiency Asian zebu ancestry regions and candidate regions with signatures of positive selection were retrieved from the *Ensemble Genes 86* database [35]. Bovine Quantitative Trait Loci (QTL) mapped on the UMD3.1 reference genome (http://www.animalgenome.org/cgi-bin/QTLdb/BT/index) intersecting with the Sheko candidate regions were also identified.

### Genetic differentiation *F*_*st*_ analysis

Genetic differentiation analysis was conducted between the Asian zebu Nelore and the African taurine N’Dama breeds using Weir and Cockerham’s *F*_*st*_ estimator [36] calculated by the hierfstat package [37] for R software. *F*_*st*_ values were estimated for each genotyped SNP and averaged over 1 Mb sliding windows overlapping by 10 kb, in which windows with a single SNP were excluded. Genomic windows in the top 1% tail of the windows *F*_*st*_ values distribution were considered as differentiated windows for further analyses. Overlapping windows were merged into candidate genomic regions.

## Results

### Asian zebu ancestry deviation on Sheko cattle genome

**Fig 1:**
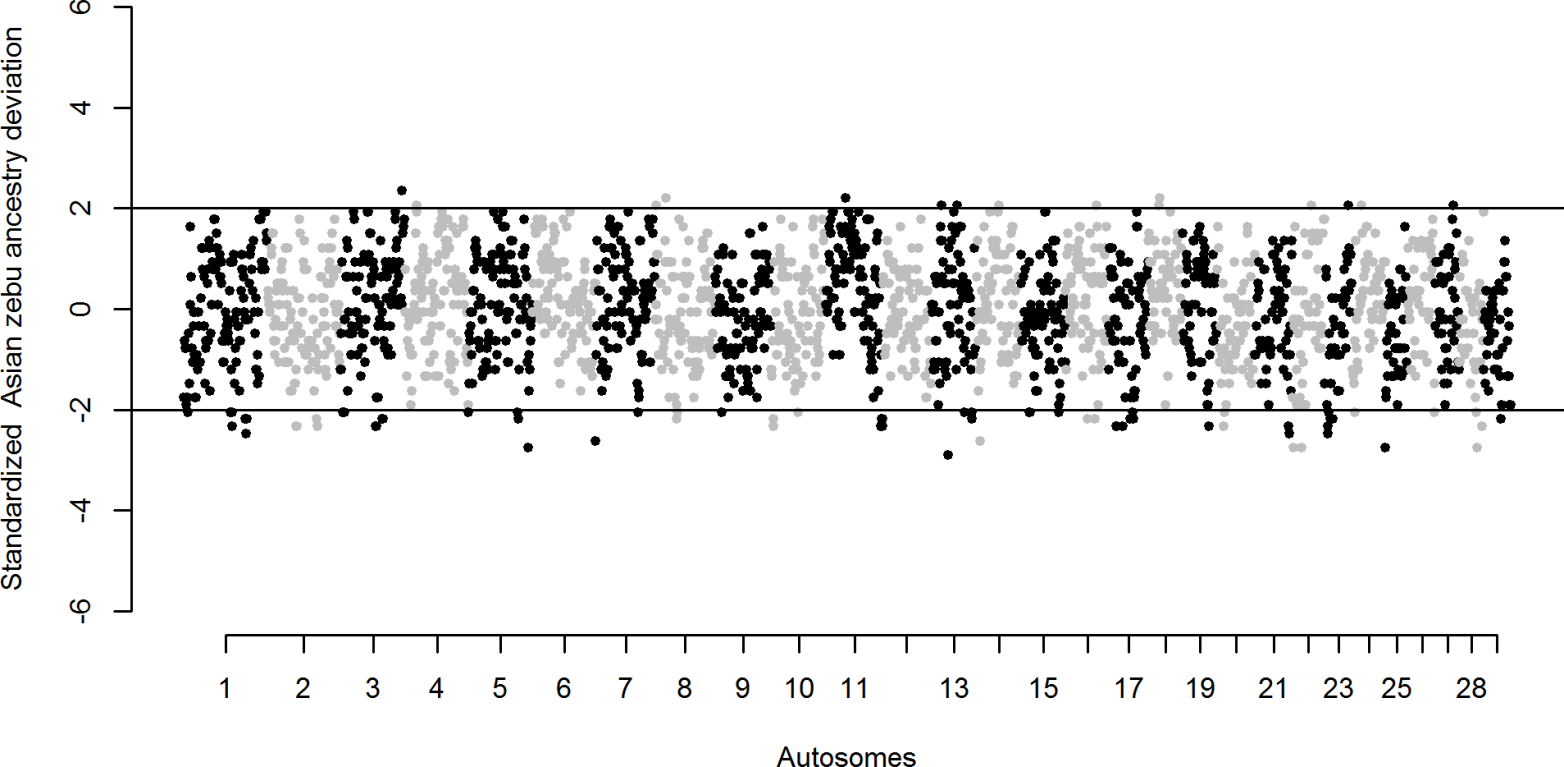
Manhattan plot of standardized Asian zebu ancestry deviation on Sheko autosomes. Sheko cattle autosomes plot showing deviation (excess/deficiency) in standardized Asian zebu ancestry in 1Mb sliding windows.

The locus-ancestry deviation analysis on the sliding 1 Mb genomic windows indicates a mean Asian zebu and African taurine ancestry proportions of 0.56 ± 0.18 and 0.44 ± 0.18, respectively. Out of the total 2,314 genomic windows, 15 of these distributed across 12 autosomes show substantial excess in Asian zebu ancestry. Whilst, 72 genomic windows distributed across 24 autosomes show substantial deficiency in Asian zebu ancestry “Fig 1 and S1 Table”.

### Candidate iHS and Rsb regions on Sheko cattle

**Fig 2:**
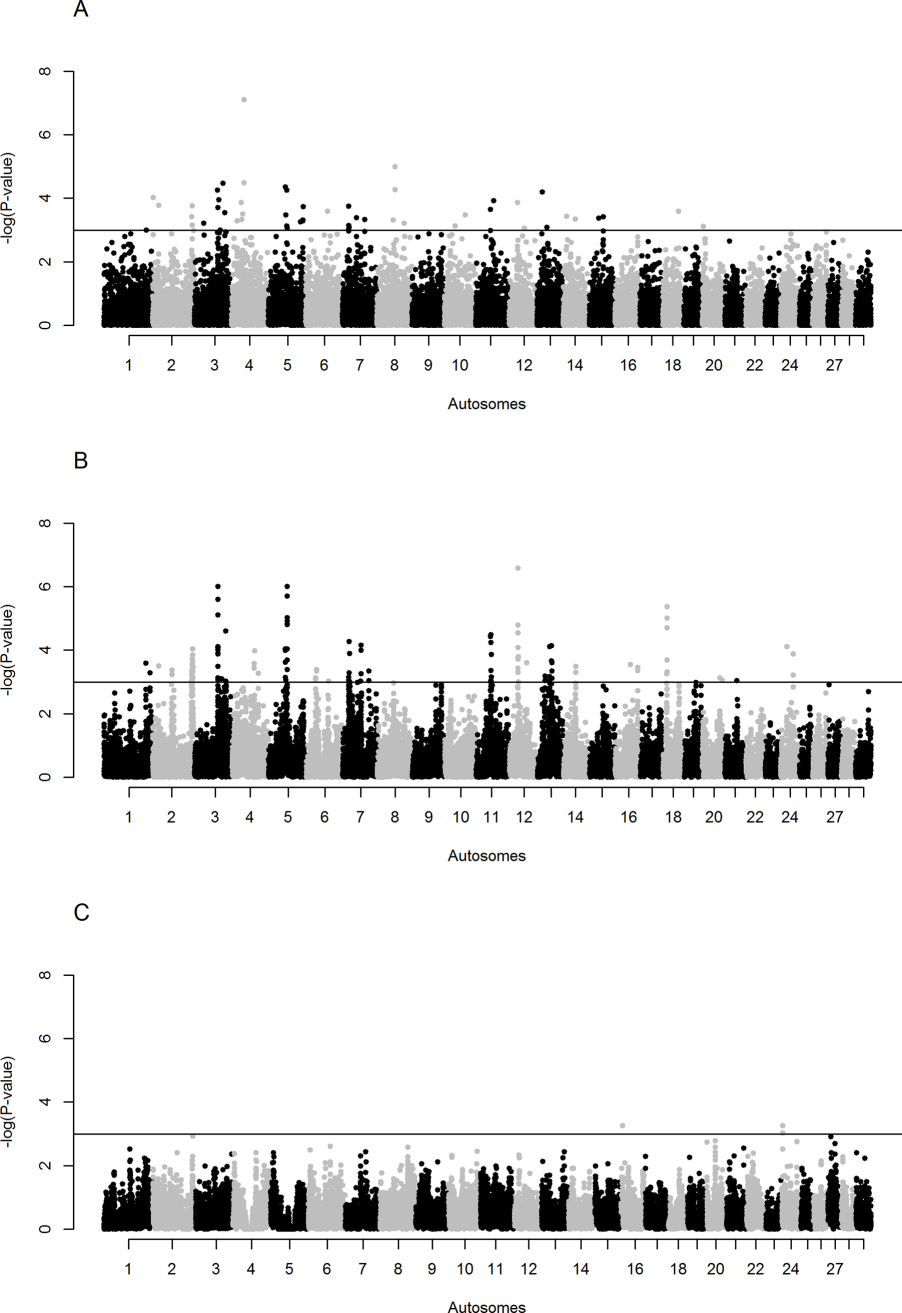
Manhattan plots of genome-wide signatures of positive selection analyses. (A) *iHS* analysis (B) *Rsb* analysis with the African taurine N’Dama cattle and (C) *Rsb* analysis with the Asian zebu Nelore cattle. The significance threshold is set at–log_10_ (two-tailed *P*-value for *iHS* analysis) and (one-tailed *P*-value for *Rsb* analysis) = 3.

The intra-population *iHS* analysis reveals nine candidate regions with signatures of positive selection across six autosomes, one on BTA 2, 7 and 8, and two on BTA 3, 4 and 5 “Fig 2A”. The inter-population *Rsb* analyses of Sheko with the N’Dama and Nelore cattle reveal 22 candidate regions with signatures of positive selection on 11 autosomes for the N’Dama comparison (one on BTA 3, 6, 14, 18 and 24; two on BTA 5, 11 and 12; three on BTA 2; four on BTA 7 and 13), and a single candidate region for the Nelore comparison on BTA 24 “Fig 2B and 2C and S2 Table”.

### Overlap among the EHH-based statistics and the locus-ancestry deviation analysis

A total of four *iHS* candidate regions on BTA 2, 3, 5 and 7 overlap with the N'Dama comparison *Rsb* candidate regions. Whilst the single *Rsb* candidate region with Nelore cattle on BTA 24 overlaps with a genomic window showing substantial deficiency in Asian zebu ancestry (BTA 24: 4.46–5.43 Mb; Table 1).

**Table 1:** Chromosomes and positions (in Mb) of overlapping candidate selected regions detected by locus-ancestry deviation, *iHS*, *Rsb* and *Fst* analyses.

Zebu deficient genome regions	His Sheko	Rsb Sheko -N'Dama	Rsb Sheko -Nelore	Fst Nelore–N’Dama
	2: 129.61–129.68	2: 129.61–130.4		
	3:79.18–79.22			3:78.2–79.5
	3: 75.83–76.12	3: 75.83–76.8		
	5: 60.58–61.4	5: 60.51–61.4		
	7: 20.66–21.6	7: 21.55–21.6		
8:40.28–41.28				8:39.6–40.8
8:42.44–43.24				8:42.5–43.5
		11:46.82–47.18		11:46.3–48.7
23:10.5–11.48				23:10.4–11.5
24: 4.46–5.53			24: 4.47–4.61	

### Functional annotation of the Sheko candidate regions

A total of 71 genes are found within substantial excess zebu ancestry regions and 721 genes within deficient zebu ancestry regions “S3 Table”. The candidate *iHS* signatures of positive selection regions have 57 annotated genes, while 85 genes are present within the N’Dama comparison *Rsb* regions, and two genes in the single Nelore comparsion *Rsb* region “S4 Table”. These genes are associated with several biological functions, such as immunity (e.g. *IL7*, *IL15*, *FCN2*, *ICOS*, *LTA4H* and *NFAM1*), fertility (e.g. *MEA1*, *CLGN* and *RXFP2*), heat tolerance (*HSPA6* and *DNAJC6*) and lactation (*PRLH*) “Table 2”.

**Table 2:** Examples of candidate genes within the candidate regions of the different analyses conducted in the study. Candidate regions are represented as (BTA: start Mb—stop Mb).

Biological role	Candidate regions	Analysis	Gene ID
**Immunity**	2:91.99–92.950	Locus-ancestry analysis*	*ICOS*
** **	5:60.51–61.4	Rsb (Sheko-N'Dama)	*LTA4H*
** **	5:112.95–113.91	Locus-ancestry analysis*	*NFAM1*
** **	11:106.24–107.04	Locus-ancestry analysis*	*FCN2*
	14:43.35–44.35	Locus-ancestry analysis**	*IL7*
** **	17:15.94–16.87	Locus-ancestry analysis*	*IL15*
**Fertility and reproduction**	12:29.03–29.72	Rsb (Sheko-N'Dama)	*RXFP2*
** **	17:15.94–16.87	Locus-ancestry analysis*	*CLGN*
** **	23:16.02–17.01	Locus-ancestry analysis*	*MEA1*
**Heat tolerance**	3:7.74–8.72	Locus-ancestry analysis*	*HSPA6*
** **	3:80.1–80.93	Locus-ancestry analysis*	*DNAJC6*
**Lactation**	3:116.99–117.96	Locus-ancestry analysis**	*PRLH*

*Zebu ancestry deficient region

**Zebu ancestry excess region

A total of 188 QTL overlap with the excess zebu ancestry regions and 706 with the deficient zebu ancestry regions “S5 Table”. Moreover, 124, 284 and eight QTL intersect with *iHS*, N’Dama comparison *Rsb* and Nelore comparison *Rsb* candidate regions, respectively “S5 Table”. These QTL are linked with different biological pathways, such as lactation (e.g. milk yield, milk fat percentage and milk protein yield), fertility (e.g. calving ease, gestation length and sperm motility), body composition (e.g. rump angle, foot angle and height) and immunity (e.g. tick resistance). Trypanotolerance-controlling QTL, identified by a cross between tolerant West African N’Dama and susceptible East African Boran cattle by [38], are found within two zebu ancestry excess, five zebu ancestry deficient and four N’Dama comparison *Rsb* regions “S6 Table”.

### Genetic differentiation regions between the Asian zebu and African taurine cattle

**Fig 3:**
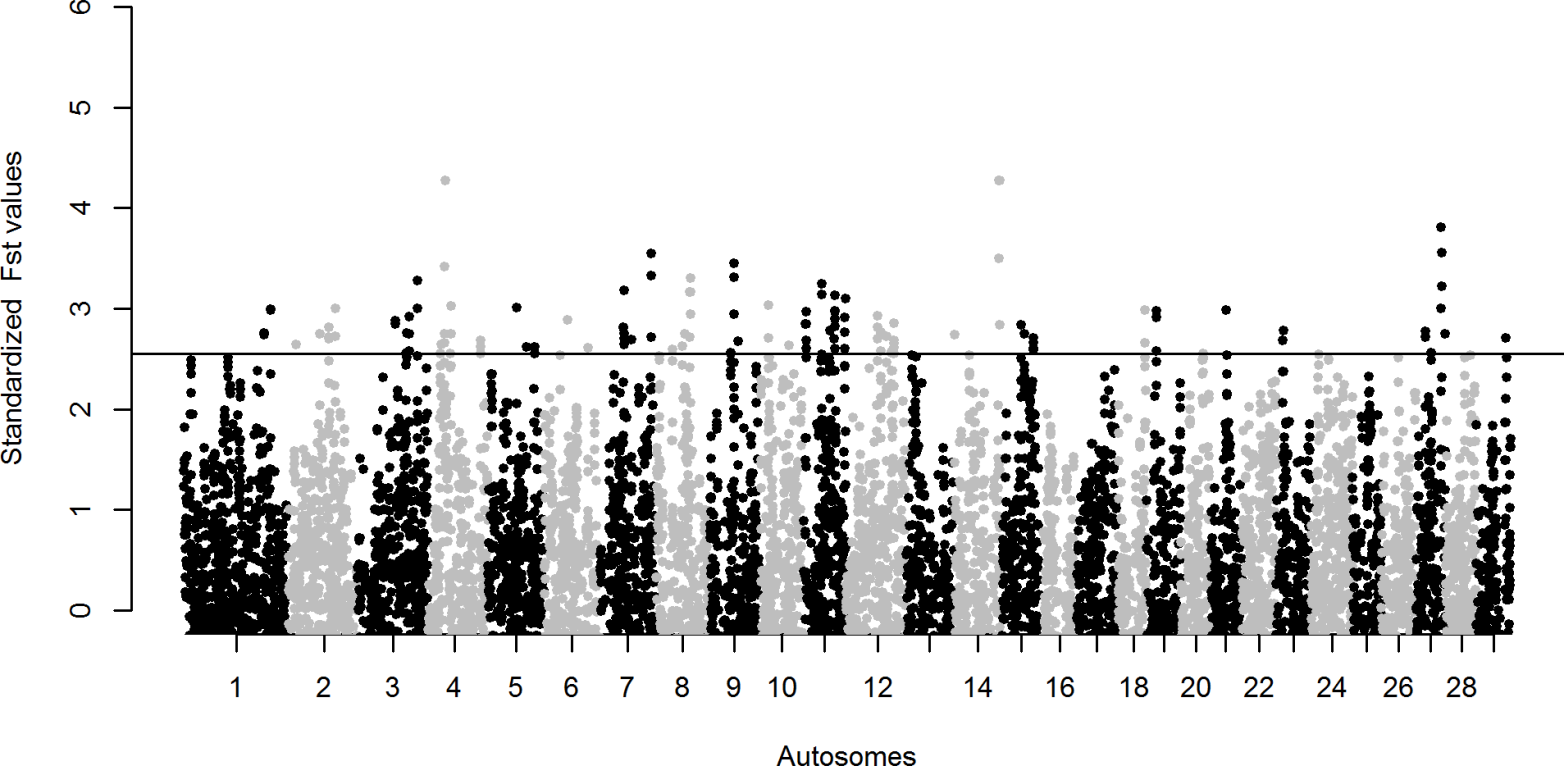
Manhattan plot of the genome-wide *Fst* analysis between the Asian zebu Nelore cattle and the African taurine N’Dama cattle breeds (1 Mb sliding window). The significance threshold is set at the top 1% of the *Fst* values distribution tail.

The mean *Fst* value of sliding 1 Mb windows between the Nelore and N’Dama cattle breeds is 0.15 ± 0.07. Upon merging genomic windows, the top 1% tail of the *Fst* values distribution contained a total of 57 regions distributed across 21 autosomes. These are considered as highly differentiated genomic regions between these two cattle breeds “Fig 3 and S7 Table”.

Two of the *Fst* regions overlap with candidate regions for signatures of selection in Sheko cattle (one *iHS* region and one N’Dama comparison *Rsb* region). Three *Fst* regions overlap with regions showing substantial deficiency in zebu ancestry in the Sheko cattle genome “Table 1”.

## Discussion

The genome of Sheko cattle was analyzed, using genome-wide medium density SNP data, to identify candidate genomic regions with signatures of adaptive introgression and positive selection. These regions were defined based on locus-ancestry deviation analysis and two EHH-based statistics (*iHS* and *Rsb*). We inferred the origin of these selection footprints as pre- or post-admixture based on genetic differentiation analysis between the two Sheko ancestral cattle breeds: N’Dama and Nelore.

### Genomic regions with signatures of adaptive introgression and natural selection

The first cattle on the African continent were of the taurine types. Subsequently, the spread of Asian zebu ancestry in the African continent from their center of domestication in the Indian subcontinent has led to various indigenous African cattle breeds with admixed Asian zebu x African taurine genomic structure [3]. The genome of these admixed cattle breeds would have been subjected to selective pressures to maximize the reproductive fitness of the crosses and their adaptability to the environmental challenges.

Adaptive introgression for advantageous zebu characteristics may be expected, while some taurine genomic regions previously selected for local adaptation would have resisted introgression. In the Sheko cattle, 87 candidate genomic regions showed substantial deviation in Asian zebu ancestry, of which 15 regions showed an excess and 72 showed a deficit of zebu ancestry, indicating candidate signatures of positive selection. Although the genome of Sheko cattle is mainly composed of zebu ancestry [12], about 83% of the candidate ancestry deviation regions showed deviation towards the taurine haplotypes. This supports the likelihood that these regions are of importance for the adaptability of Sheko cattle to the local environment. Interestingly, five of these zebu ancestry deficient regions overlap with five trypanotolerant QTL, while two of these regions with excess of zebu ancestry overlap with two trypanotolerant QTL. This is not surprising as it has been shown that both regions of zebu and taurine origin may contribute to the trypanotolerance characteristic of West African N'Dama and East African Boran crossbreeds [38].

Moreover, the two different EHH-based analyses, *iHS* and *Rsb*, identified 32 candidate regions with signatures of positive selection in Sheko cattle (nine regions for *iHS*, 22 regions for *Rsb* Sheko—N’Dama comparison, one region for *Rsb* Sheko—Nelore comparison). The *Rsb* Sheko—N’Dama analysis results support selection pressures on zebu haplotypes, whilst, the *Rsb* Sheko–Nelore analysis indicates that the taurine haplotypes within this region are the target of selection. These results require further investigation and validation using full genome sequence data of Sheko cattle and the ancestral cattle breeds.

### The confounding effect of the natural demographic history and selection

Demographic population processes, such as migration and the associated gene flow and genetic drift, also shape the genome diversity of livestock populations and may lead to similar signals as natural selection at the genome-wide level [39]. This will be the case in pure breeds as well as in admixed populations. Concerning the latter, taurine or zebu haplotypes may have become fixed following random segregation of alleles subsequent to admixture. However, it could be argued that in the absence of selection such taurine or zebu fixed regions in the crossbreed will show sequence diversity. The overlap between four *iHS* and *Rsb* candidate regions, and between a single zebu-deficient region with an *Rsb* candidate region “Table 1” supports the role of selection pressures, and not natural demographic processes, in shaping the genomic pattern of these regions. This low level of overlap between *iHS* and *Rsb* selection and introgression may be a consequence of a lack of power in the analyses performed here. A caveat of the *iHS* analysis is that it will not identify a selected haplotype which has reached or is close to reaching fixation, while the *Rsb* analysis cannot identify signatures of selection for haplotypes that are under selection in both breeds being compared [39]. The methods we have applied target different selection timeframes, with the ancestry deviation approach targeting recent post-admixture selection, while the EHH-based statistics identify much older signals of selection [40], and as such we would not expect to observe significant overlap across the results. Indeed, a study on the admixed Swiss Fleckvieh cattle breed, which is a composite of Simmental and Red Holstein-Friesian cattle breeds, also resulted in little overlap when applying the same approaches [22]. Increasing the sample size and density of the SNP data, for example through whole-genome sequencing, will greatly improve the power of these tests, enabling a more robust investigation into signatures of selection in Sheko cattle. Nonetheless, 24 candidate regions (three zebu excess, 11 zebu deficient, two *iHS* and eight Sheko—N’Dama *Rsb* regions) do overlap with candidate genomic regions under positive selection reported in previous studies on indigenous African cattle breeds such as the East African Shorthorn Zebu [16, 17], and the Butana and Kenana cattle [18], as well as commercial cattle breeds, Murray Grey, Shorthorn and Charolais [20].

### Functional annotation of the candidate regions

Several genes and QTL associated with different biological pathways, e.g. immunity, fertility and reproduction, heat stress, and the dairy production characteristics of Sheko cattle, have been identified within the candidate selected regions. These genes and QTL might be related to the adaptation of Sheko cattle to the local environment and hence can be considered as targets of natural selection in Sheko cattle. These cattle are known to be tolerant to different endemic parasitic diseases [11, 41], and so the immunity-related genes within the candidate regions identified (e.g. *LTA4H*, *IL7*, *IL15*, *FCN*, *LTA4H* and *NFAM1*) are potential targets of natural selection. An immunity-related gene identified in a Sheko—N’Dama *Rsb* candidate region on BTA 5 is leukotriene A-4 hydrolase (*LTA4H*). This gene is associated with immune response regulation and inflammation response in mammals [42]. *LTA4H* was also been identified within a candidate region of positive selection in East African shorthorn zebu cattle (EASZ) from Kenya [16]. Two interleukin genes (*IL7* and *IL15*) were identified in zebu-excess and zebu-deficient regions on BTA 14 and BTA 17, respectively. Interleukin-7 is an important cytokine involved mainly in the early development of B- and T-cells [43]. Whilst, Interleukin-15 mediates the activation of natural killer cells [44].

Genes related to fertility and reproduction are hotspots of selection in indigenous cattle breeds living in tropical environments. The relaxin/insulin-like family peptide receptor 2 (*RXFP2*) gene is present in a Sheko—N’Dama *Rsb* candidate region on BTA 12. This gene is involved in the testicular descent development [45], and has also been found to be under selection in two different tropical-adapted admixed cattle population; EASZ [16, 17] and Creole cattle [46]. The calmegin (*CLGN*) gene, located in a zebu-deficient candidate region on BTA 17, is a testis-specific Ca^+2^-binding protein involved in mediating the binding between eggs and sperms during fertilization [47]. The male-enhanced antigen-1 (MEA1) gene found within a zebu-deficient candidate region on BTA 23 is expressed mainly in spermatids indicating a possible role in late stages of spermatogenesis [48].

The agro-ecological zone of the sheko is classified as warm and humid to peri-humid, characterized by a mean annual temperature of 22.6°C and annual rainfall from 1200 to 2200 mm [49]. In such an environment tolerance to heat and humidity will be advantageous. *HSPA6* and *DNAJC6* genes are both found within zebu-deficient candidate regions on BTA 3. The heat shock protein family A member 6 (*HSPA6*) is a member of the heat shock protein (Hsp70) family which protect cells from lethal damage caused by heat stress through maintaining the folding of newly synthesized proteins and assembly of multi-protein complexes [50]. DNAJC6 acts as a co-factor for Hsp70 family to mediate their cellular function [51]. Members of these two gene families have also been found previously to be under selection in EASZ [16, 17].

Sheko cattle are considered a breed with good dairy potential. Several dairy production-related QTL (e.g. milk yield, milk fat percentage and milk protein yield) overlap the candidate regions identified, including the prolactin releasing hormone (*PRLH*) gene which overlaps with a zebu ancestry-excess region. A study in African zebu cattle also identified a candidate selection peak at the prolactin releasing hormone (*PRLH*) gene [19]. In addition, it has been shown that mutation at prolactin (*PRL*) and its receptor (*PRLR*) genes have an impact on thermoregulation and hair morphology [52]. The prolactin pathway might therefore have been selected in Sheko cattle both in relation to milk production and heat tolerance.

### Origin of selection: Pre- or post-admixture?

Highly differentiated genomic regions between the ancestral populations of an admixed population may indicate ancient signatures of selection prior to admixture [22, 24]. We found overlaps between three zebu ancestry deficient regions and a Sheko—N’Dama *Rsb* candidate region with highly differentiated regions between Nelore and N’Dama cattle. While the former suggest signals of ancient selection within African taurine prior to admixture, the later suggest an ancient zebu selected region. However, these results require further validation using a higher density genome-wide SNP chip, such as the Illumina BovineHD Genotyping BeadChip, and/or full genome sequence data.

## Conclusion

In this study we employed genome-wide medium density SNP data to investigate the genome of Sheko cattle for regions with signatures of adaptive introgression and positive selection. Several candidate regions were identified showing excess and deficiency in zebu ancestry and unusual extended haplotype homozygosity. These regions are associated with different biological traits such as immunity, reproduction, heat tolerance and lactation. Some of these selection signals are likely to be a result of ancient selection prior to the admixture of the ancestral African taurine and Asian zebu breeds. Our findings contribute towards improving our understanding the genome of the Sheko cattle breed, and can inform breeding programmes to enhance the productivity and sustainability of the indigenous African cattle in their native environment. However, further validation and investigation using a larger sample size and high-resolution data, such as that from a high-density SNP array or full genome sequence data, is required to better characterize the favorable haplotypes or variants under selection.

## Supporting information

S1 Fig Histograms showing the distribution of the (A) standardized *iHS* values, (B) standardized Sheko—N'Dama *Rsb* values and (C) standardized Sheko—Nelore *Rsb* values.
(TIFF)

S1 Table Genomic windows on Sheko cattle showing substantial excess and deficiency in Asian zebu ancestry and overlapping with signatures of selection in other cattle breeds from previous studies. (XLSX)

S2 Table Candidate signatures of selection regions identified by the *iHS* and *Rsb* analyses and overlapping with signatures of selection in other cattle breeds from previous studies. (XLSX)

S3 Table Genes within the substantial excess and deficient zebu ancestry regions. (XLSX)

S4 Table Genes within the candidate signatures of selection regions identified by *iHS* and *Rsb* analyses. (XLSX)

S5 Table Quantitative trait Loci (QTL) within the candidate regions defined by the locus-ancestry deviation, *iHS* and *Rsb* analyses. (XLSX)

S6 Table Trypanotolerance-controlling QTL overlapping zebu-ancestry excess and deficient regions and Sheko-N'Dama Rsb candidate regions. (XLSX)

S7 Table Candidate genetically differentiated regions between Nelore and N'Dama cattle on the top 1% of the Fst values distribution. (XLSX)
