# Supplementary figures and images for: Genomic signatures of adaptive introgression and environmental adaptation in the Sheko cattle of southwest Ethiopia
Source: PLoS One. 2018 Aug 16;13(8):e0202479. doi: 10.1371/journal.pone.0202479 (PMC6095569; doi:10.1371/journal.pone.0202479)

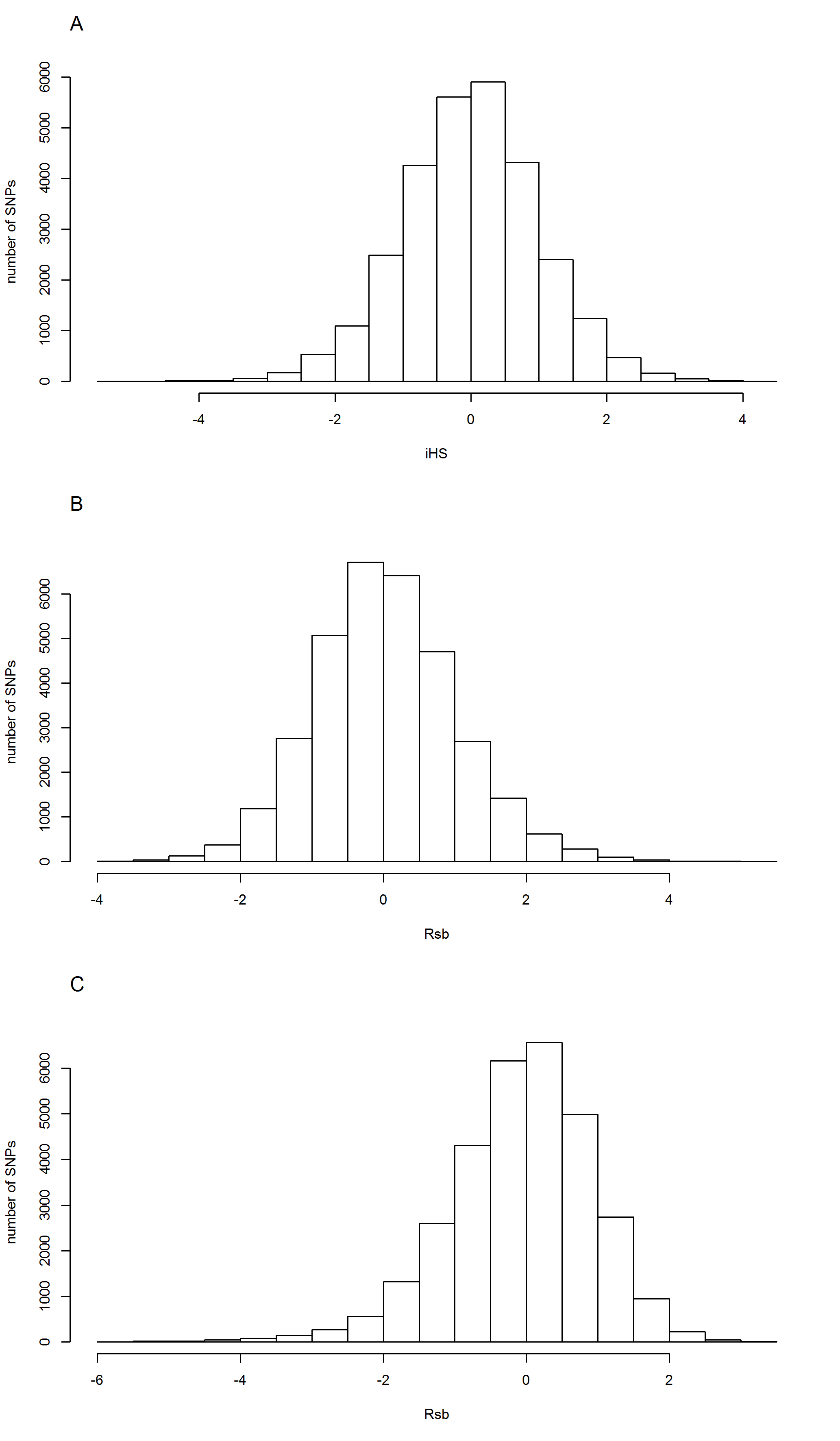

Supplement: S1 Fig — Histograms showing the distribution of the (A) standardized iHS values, (B) standardized Sheko—N'Dama Rsb values and (C) standardized Sheko—Nelore Rsb values. (TIFF) [file pone.0202479.s001.tiff]
